# Genome-Wide Analysis of Alternative Splicing during Host-Virus Interactions in Chicken

**DOI:** 10.3390/v13122409

**Published:** 2021-12-02

**Authors:** Weiwei Liu, Yingjie Sun, Xusheng Qiu, Chunchun Meng, Cuiping Song, Lei Tan, Ying Liao, Xiufan Liu, Chan Ding

**Affiliations:** 1 Shanghai Veterinary Research Institute, Chinese Academy of Agricultural Sciences, Shanghai 200241, China; liuweiwei@shvri.ac.cn (W.L.); sunyingjie@shvri.ac.cn (Y.S.); xsqiu1981@shvri.ac.cn (X.Q.); mengcc@shvri.ac.cn (C.M.); scp@shvri.ac.cn (C.S.); tanlei@shvri.ac.cn (L.T.); liaoying@shvri.ac.cn (Y.L.); 2 School of Veterinary Medicine, Yangzhou University, Yangzhou 225009, China; xfliu@yzu.edu.cn; 3 Jiangsu Co-Innovation Center for Prevention and Control of Important Animal Infectious Diseases and Zoonoses, Yangzhou University, Yangzhou 225009, China

**Keywords:** alternative splicing, ALV, NDV, IBDV, AIV, chicken, virus-host interaction

## Abstract

The chicken is a model animal for the study of evolution, immunity and development. In addition to their use as a model organism, chickens also represent an important agricultural product. Pathogen invasion has already been shown to modulate the expression of hundreds of genes, but the role of alternative splicing in avian virus infection remains unclear. We used RNA-seq data to analyze virus-induced changes in the alternative splicing of Gallus gallus, and found that a large number of alternative splicing events were induced by virus infection both in vivo and in vitro. Virus-responsive alternative splicing events preferentially occurred in genes involved in metabolism and transport. Many of the alternatively spliced transcripts were also expressed from genes with a function relating to splicing or immune response, suggesting a potential impact of virus infection on pre-mRNA splicing and immune gene regulation. Moreover, exon skipping was the most frequent AS event in chickens during virus infection. This is the first report describing a genome-wide analysis of alternative splicing in chicken and contributes to the genomic resources available for studying host–virus interaction in this species. Our analysis fills an important knowledge gap in understanding the extent of genome-wide alternative splicing dynamics occurring during avian virus infection and provides the impetus for the further exploration of AS in chicken defense signaling and homeostasis.

## 1. Introduction

Alternative pre-mRNA splicing (AS) is an essential mechanism for generating transcriptome plasticity and proteome diversity in eukaryotes [1]. The alternative splicing variants of a transcript often have different structures, functions, sub-cellular localizations and stability [2]. This process is carried out by the spliceosome, which consists of small nuclear RNAs (snRNA) and approximately 180 different proteins [3]. The spliceosome removes introns from a transcribed pre-mRNA through recognition of consensus 5′ and 3′ splice sites, a branchpoint, and a polypyrimidine tract. The conserved consensus sequences found at the 5′ and 3′ splice sites and branch site in metazoans is GURAGU, YAG and YNCURAC [4]. The regulation of AS depends on cis-acting sequences located in exonic and intronic regions, called splicing enhancers or silencers. Trans-acting modulators also play a significant role, including serine-arginine repeat (SR) proteins and heterogeneous nuclear ribonucleoprotein particle (hnRNP) protein families. These trans actors can recognize cis-acting sequences and then activate or repress the assembly of the splicing complex [5,6]. Changes in the abundance, localization, and activity of these regulators in various cells and in different environments have genome-wide influences on the splicing patterns of genes. AS often incorporates premature stop codons in alternate transcripts that result in transcripts which are degraded through a process called nonsense-mediated decay (NMD) [7]. It has been estimated that 90% to 95% of genes in the human genome are alternatively spliced, with exon-skipping events as in the predominant AS type [8,9]. Changes in alternative splicing can be the cause or the consequence of human diseases and a subset of alternative splicing events have been identified to regulate development, tissue identity, pluripotency and tumor proliferation [10,11,12,13,14,15].

Chicken (Gallus gallus) is an important model organism that bridges the evolutionary gap between mammals and other vertebrates and serves as the main laboratory model for over 9600 extant avian species [16]. Chicken is also a model animal for studying immunity and development, and represents an important agricultural animal with significant economic value [17]. Compared with humans, the percentage of common AS events is approximately 20% between human and chicken [18]. The major splice site motifs in chicken AS genes were found to be GT-AG, which was consistent with human and mouse but the splice site motifs GC-AG and AT-AC in chicken AS genes were not often found in human and mouse [19].

Avian pathogens, such as avian leukosis virus (ALV), infectious bursal disease virus (IBDV) and avian influenza virus (AIV), cause thousands of poultry diseases and deaths annually, resulting in significant economic loss to the poultry industry [20,21,22]. Due to its superior reproduction ability, the Leghorn chicken plays an important role in the commercial egg supply market [23]. Unfortunately, it has very little ability to resist diseases. Fayoumi is an indigenous chicken breed that originated in Egypt, which is well known for its strong resistance to various pathogens and its tolerance to unfavorable environmental conditions [24,25]. To discover the underlying mechanisms of the distinct phenotypic differences in avian influenza resistance, Leghorn and Fayoumi have previously been compared at the DNA, gene expression, and methylation levels [24,26,27]. Although Fayoumi chickens are much less susceptible to diseases, they do not show complete immunity, implying that their resistance mechanism needs to be further investigated.

During viral infection, the host often recognizes the invading pathogen through pattern recognition receptors (PRRs) and launches robust immune responses through a series of signal cascades to resist invasion [28,29]. In order to counter host defenses, pathogens use a variety of strategies to evade recognition or inhibit host responses. For example, Hit and run viruses evade immune destruction by infecting new hosts and rarely persist. Hit and stay viruses evade immune control by sequestration, blockade of antigen presentation, cytokine escape, evasion of natural killer cell activities, escape from apoptosis, and antigenic change [30]. At the cellular level, alternative splicing represents one means of regulating host defenses. Pathogen invasion is known to result in the differential expression of hundreds of genes affecting metabolism, signal transduction, transcriptional regulation, posttranscriptional gene silencing, protein degradation, and other cellular processes [31,32,33,34,35].

To date, there have been a small number of studies which employed transcriptome-wide microarray or RNA-seq to analyze AS events in virus-infected cells. Studied viruses include herpesviruses, reoviruses, dengue viruses (DENV), Zika virus, and influenza viruses [36,37,38,39,40,41]. These studies revealed several hundred host genes that have altered mRNA splicing upon infection. However, genome-wide analysis of AS in response to viral infection in chicken is yet to be carried out. We recently reported the alteration of host transcriptome profiles in chicken during infection by the highly virulent Newcastle Disease Virus (NDV) Herts/33 strain or the nonvirulent LaSota strain, which is a member of the family Paramyxoviridae (genus Avulavirus in subfamily Paramyxovirinae) [42]. Newcastle disease virus (NDV) infection presents as a serious respiratory disease and is one of the most important infectious diseases of poultry.

In this study, we used high-throughput mRNA sequencing (RNA-seq) data to detect transcriptome changes in chickens during viral infection in order to investigate the impact on splicing regulation. In addition to discovering thousands of previously unannotated transcripts, we identified AS events in key immune-related gene clusters encoding protein kinases, receptors, transcription factors, proteases and nucleic acid binding proteins. Taken together, our results significantly expand the current understanding of virus-induced alternative splicing in chicken and open up a number of new avenues for future exploration on AS in chicken.

## 2. Materials and Methods

### 2.1. Data Sources

RNA-seq raw sequence data of the CEF cells infected with Newcastle Disease Virus were obtained from our previous study [42]. Other RNA-seq data, such as avian influenza virus (H5N3), infectious bursal disease virus (IBDV) and avian leukemia virus (ALV) were retrieved from the NCBI Short Read Archive database under the accession numbers SRP041937, ERP006921 and ERP017744 [27,43]. RNA-seq data from tissues infected with NDV, such as lung, spleen, trachea and the Harderian gland were also retrieved from the NCBI Short Read Archive database under the accession numbers ERP024044, ERP023964, ERP021317 and ERP104372 [44,45,46,47].

### 2.2. Read Alignment to the Reference Chicken Genome and Gene Expression Estimation

Chicken genome sequences and annotation were downloaded from Ensembl (Gallus_gallus-5.0, http://asia.ensembl.org/Gallus_gallus/Info/Index (accessed on 23 November 2021)). After removing the low-quality reads and trimming the adapter sequences using FASTQC tools, the remaining reads from each sample were mapped to the reference chicken genome using the Tuxedo RNA-seq analysis pipeline [48,49,50]. The Tuxedo pipeline was comprised of TopHat2 (read mapping) (version 2.0.1), Cufflinks2 (transcript assembly) (version 2.2.0), Cuffmerge2 (transcript model merging) and Cuffdiff2 (differential gene and transcript expression, and differential splicing analysis) (v2.1.1). These programs were run with default parameters.

### 2.3. Putative Transcript Assembly and AS Event Identification

After the reads were aligned to the genome, transcript construction for each sample was performed using Cufflinks [50]. Four coding potential analysis software packages were employed, including CNCL, phloCSF, CPC and Pfam. All software was run using the default parameters. Transcripts that were predicted to have coding potential by all four tools were used as the candidate set of mRNAs. Chromosome-level visualization of read densities was performed using Seqmonk tools (Babraham Bioinformatics) and the Integrated Genome Viewer (Broad Institute) [51]. Sashimi plots were constructed using the MISO tools framework [52,53]. RNA-seq read densities were plotted along the splice junctions to visualize gene structure and read coverage.

We employed ASTALAVISTA to analyze the various types of AS events prevalent in CEF cells in vivo and in vitro after infection with NDV and other viruses [54]. Five main types of AS events, including Exon Skipping (ES), Alternative 5′Donor Site (A5SS), Alternative 3′Acceptor Site (A3SS), Intron Retention (IR) and other AS events were analyzed.

### 2.4. Alternative Splicing Landscapes and Differential Splicing Analysis 

To quantify genes that were differentially spliced during NDV infection, Cuffdiff (v2.1.1) was used to calculate the isoform abundances of all coding genes. Differential expression analysis was also performed using Cuffdiff, with an adjusted *p* < 0.05 used as a cutoff for significant differential expression.

### 2.5. Functional Enrichment, Clustering, and Isoform-Level Differential Expression Analysis

To identify functional terms enriched among the differentially spliced genes, we performed set enrichment analysis using the PATHER. GO enrichment analysis on genes that were differentially expressed or spliced during viral infection, employing a χ^2^ test for significance evaluation, followed by FDR correction (FDR < 0.05) to enrich for statistically significant functional terms.

To identify genes that are alternatively spliced as a result of infection, the differential expression of protein coding genes with at least two transcripts was analyzed using Cuffdiff, followed by FDR correction (FDR < 0.05). These differentially spliced genes were then analyzed by GOEAST for enrichment.

The alternative splicing of the genes associated with immune functions based on KEGG, GO and PANTHER annotations were selected for further analysis. Hierarchical clustering of the differentially spliced gene clusters encoding protein kinases, protein receptors, transcription factors, proteases and nucleic acid binding proteins was then performed using R packages pheatmap (version 1.0.10).

### 2.6. 3′ UTR Analysis

The FPKM values of transcript 3′ UTRs were calculated by the Stringtie software and the relationship between the length of 3′ UTR and the corresponding FPKM in virus infection sample was analyzed. Dynamic APA (alternative polyadenylation) in infected versus uninfected samples was assessed by the DaPars (De novo identification of dynamic APA) algorithm [54]. To explain the localized read density changes, DaPars employs a linear regression model to determine the optimal fit point. Alignment files were converted to wig file format using RSEM software. PDUI (percentage distal usage index) differences of 0.3 with FDR < 0.05 were considered significant. The filtered result was visualized using integrated genomic viewer software. The relationship between the PDUI score and the fold change of transcripts in virus infection was also visualized.

### 2.7. Spliceosome Analysis 

The list of genes involved in splicing events in chicken were downloaded from the spliceosome database and analyzed for differential expression [55]. Volcano plots of these genes were generated in R using the ggplot package, with downregulated genes highlighted in blue and upregulated genes highlighted in red, with an FDR of less than 0.05. Spliceosome transcripts corresponding to the differentially expressed genes with a log2 fold change greater than 1 between the control and infected groups were displayed in a heatmap, which was generated in R using the ggplots package. The relationship between length and fold change was analyzed for all genes associated with the spliceosome. A list of genes belonging to different stages in the spliceosome assembly and activity were queried in the STRING database for functional associations, with an interaction network built using Cytoscape (version 3.6.1).

## 3. Results

### 3.1. Mapping Transcriptome Data to the Reference Chicken Genome

In a previous study, we reported comprehensive transcriptome changes during NDV infection in chicken embryo fibroblast (CEF) cells [42]. To further understand the isoform-level mRNA abundances and AS events altered during virus infection, we re-analyzed our transcriptome data with a focus on isoform-level quantification.

Primary chicken embryo fibroblast (CEF) cells were infected with NDV, using Herts/33 and LaSota viruses at time 0 at multiplicity of infection (MOI) 1 and then harvested at 12 h post infection. RNA was isolated from the control, Herts/33- and LaSota-infected CEF cells at 12 hpi and was subjected to 125 base paired-end Illumina HiSeq2500 sequencing, followed by quality trimming and filtering. High-quality reads were retained and mapped to the Gallus gallus reference genome (Gallus_gallus-5.0, http://asia.ensembl.org/Gallus_gallus/Info/Index (accessed on 23 November 2021)) using the Tophat2 program from the Tuxedo pipeline. Transcript assembly, differential expression analysis, and global splicing analysis were performed using Cufflinks2, Cuffmerge2, Cuffdiff2, and CummeRbund programs [50]. A split-read cutoff ≥10 was used to indicate evidence for splice junction reads.

In order to assemble novel transcripts and analyze differential isoform and gene expression, we used the Tuxedo analysis pipeline, which employs a robust isoform deconvolution method for estimating transcript expression. Novel transcripts were then filtered to remove low abundance isoforms, which could be the result of sequencing processing intermediates or incorrect transcript assembly. In total, 293 million reads were mapped to the reference genome Gallus_gallus-5.0. Mapping resulted in 85% to 87% overall alignment rates for control samples and an 82% overall alignment rate for LaSota samples. Compared with the control and LaSota samples, the alignment rate of the Herts/33 sample was approximately 67% on average (Table 1). Approximately 91% of these reads mapped uniquely to only one genomic locus, attesting to the high quality of both the sequencing reads and the reference genome (Table 1). Global inspection of isoform-level expression, normalized to fragments per kilobase of transcript per million mapped fragments (FPKM), was performed using the CummeRbund program [49].

**Figure 1:**
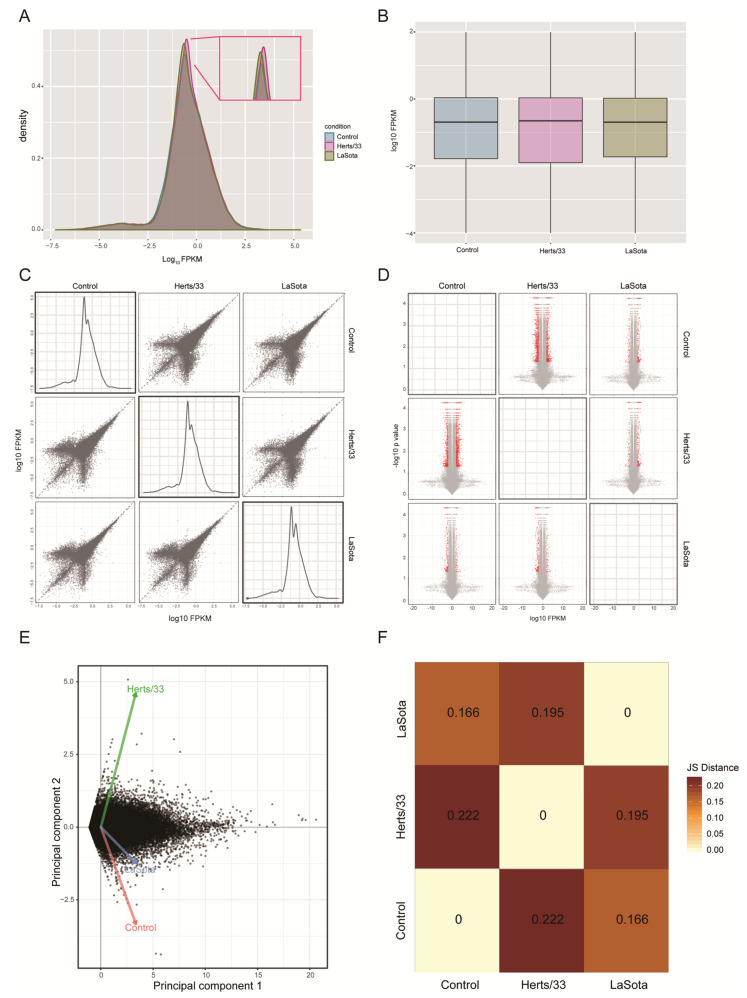
Global analysis of isoform expression in control and NDV-infected CEF cells. (**A**) Expression box plots showing density differences in NDV-infected samples (Herts/33 or LaSota) compared with control. The inset shows the expression density differences among the samples. (**B**) Expression scatter matrix in control, Herts/33 and LaSota-infected samples. (**C**) Expression fold-change volcano matrix in control, Herts/33 and LaSota-infected samples. A pairwise scatter matrix generated using the csScatterMatrix method of the CummeRbund tools to enable easy visualization of all the possible comparisons [49]. (**D**) The normal distribution of the expression data and significantly greater number of differentially expressed transcripts in virus-infected samples (Herts/33 or LaSota) compared with the control. Red indicates significant upregulation and grey indicates no significant upregulation (*p* < 0.05). (**E**) Principal component analysis of control, Herts/33 and LaSota-infected samples using a Jensen-Shannon distance matrix. The principal component plot was generated using the PCA plot wrapper of CummeRbund tools with log transformed expression estimates [34]. (**F**) Clustering and positive correlation between NDV-infected samples (Herts/33 or LaSota) and control. Together, these analyses attest to the robustness of the Gallus gallus transcriptome analysis, revealing little systematic bias among the individual samples [56], as well as enabling identification of the significant differences present among the control and virus-infected samples at genomic scale.

Density and box plots of isoform-level expression (log10 FPKM) revealed a normal overall distribution of the data with little systematic bias among control, Herts/33 and LaSota gene expression (Figure 1A,B). A large number of transcripts were upregulated in the virus-infected sample compared with the control sample, with Herts/33 showing the most changes overall (Figure 1D and Table S1). These broad patterns were indicated by positive log10 FPKM and log2 fold changes in the pairwise scatter matrix plots and volcano matrix plots, respectively (Figure 1C,D and Table S1). Multivariate clustering and statistical relationships among control, Herts/33 and LaSota samples were analyzed using principal component analysis (Figure 1E,F). Jensen–Shannon distances further indicated significant clustering of Herts/33 and LaSota datasets when compared with control. Taken together, these results indicated the Herts/33 sample had greater change at the transcriptome level than the LaSota sample when compared with the control, which was consistent with our previous transcriptome analysis [42].

The annotated Gallus gallus reference genome (Gallus gallus-5.0) contains 18,346 protein-coding genes and 30,252 transcripts (Tables S2 and S3). After transcript discovery, novel transcripts were filtered to remove low abundance isoforms, which could be the result of sequencing or assembly errors. After filtering, the merged Gallus gallus gene annotation contained 32,467 transcripts in 17,434 gene loci (Tables S4 and S5). Of these, 1928 (11%) genes and 8076 transcripts (25%) were previously unannotated but were expressed at a level of at least 2 FPKM in at least one virus-infected sample (Tables S6 and S7).

**Figure 2:**
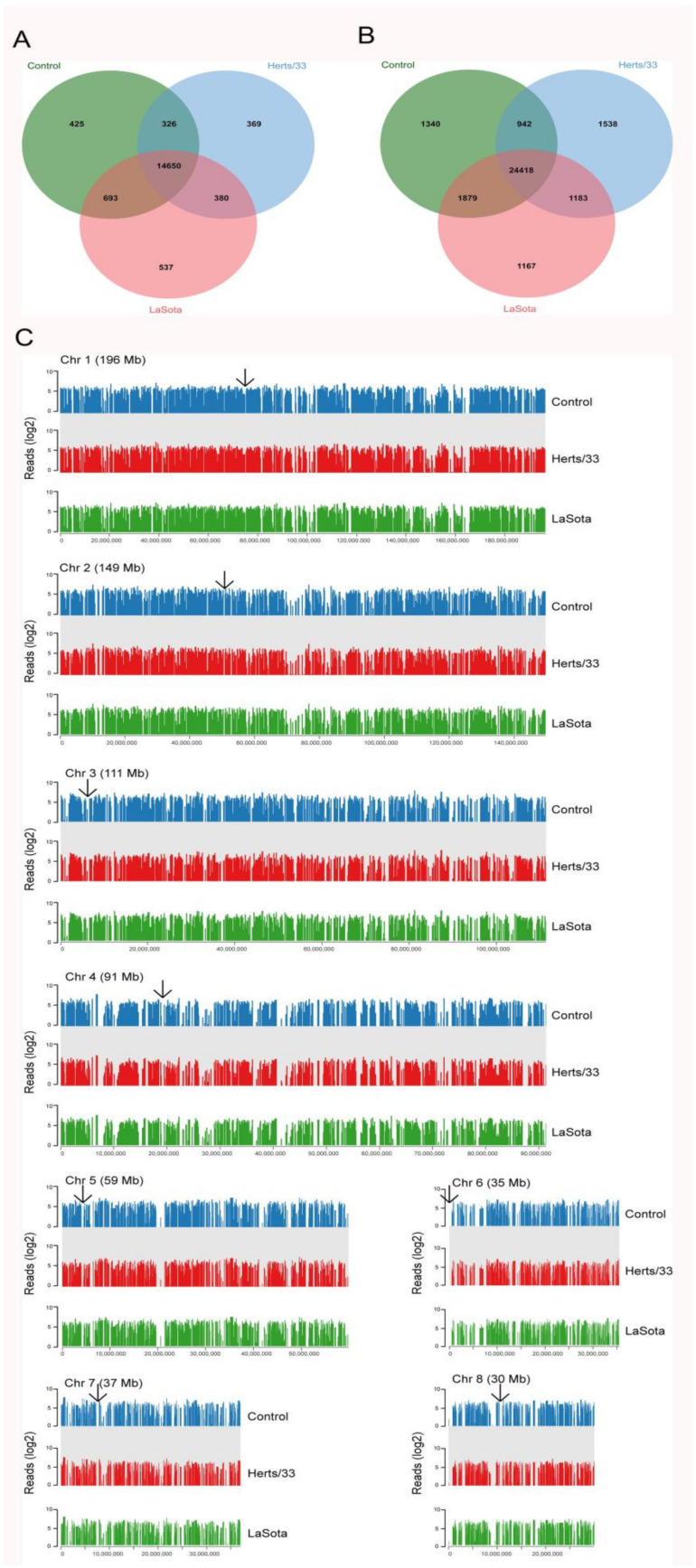

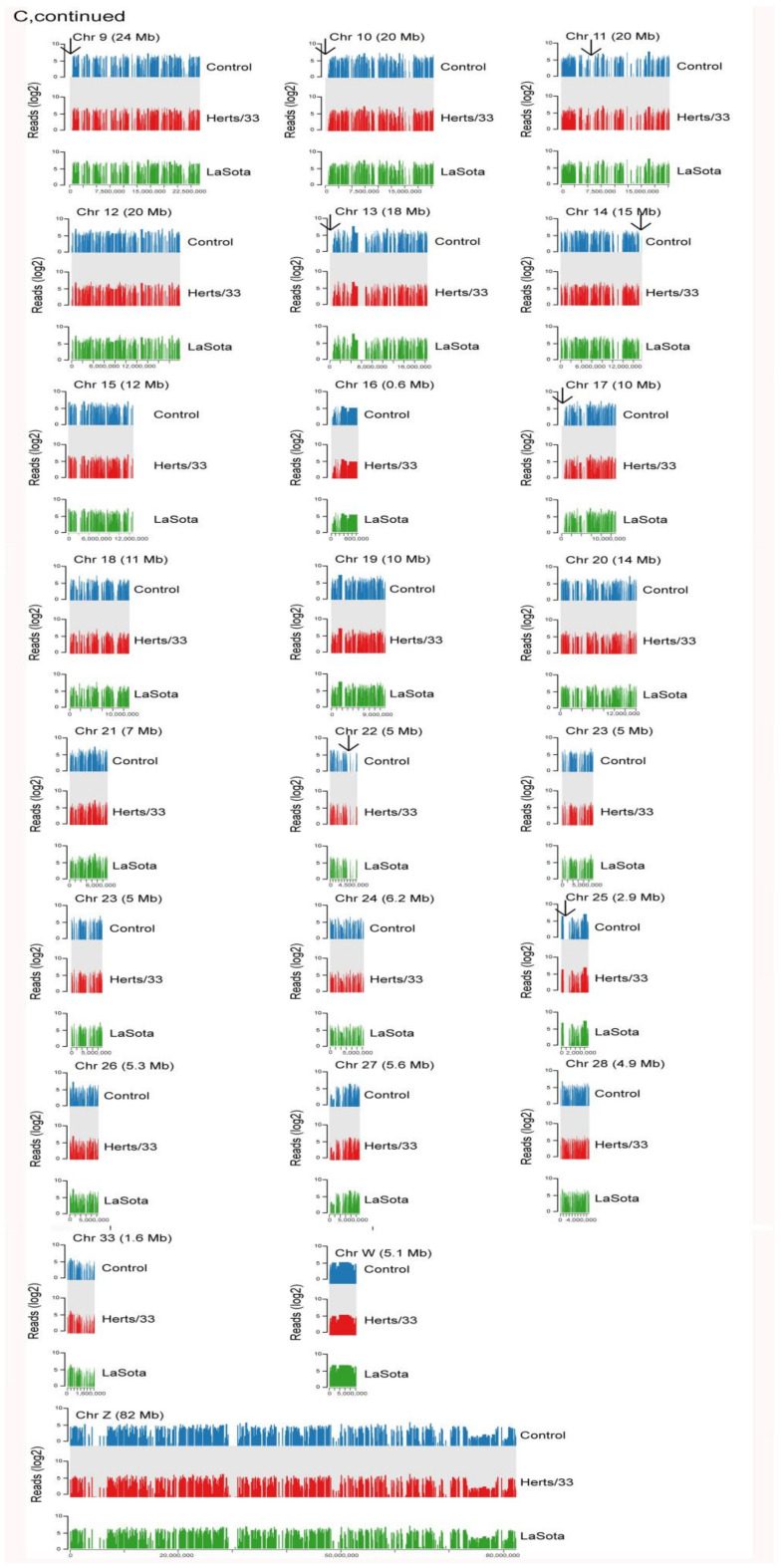
Isoform-level transcriptome maps of control and NDV-infected CEF cells. (**A**) Venn diagrams of the expressed genes and (**B**) their corresponding transcripts with at least 2 FPKM expression among the control, Herts/33-, and LaSota-infected CEF cells. (**C**) Read densities mapped against Gallus gallus chromosomes in the control, Herts/33- and LaSota-infected CEF cells show extensive transcriptional activity throughout the genome. The arrowheads indicate centromeric regions. Read densities (log_2_ RPM) were quantitated and plotted against reference chromosome models (Gallus_gallus-5.0) using Seqmonk tools.

Transcripts which were expressed in at least one sample and coded “j” or “=” by the cufflink’s assembler were selected for statistical analysis, including 17,373 genes and 32,377 transcripts (Figure 2A,B). The genome-wide distribution of expression densities in control samples was similar to that in Herts/33 and LaSota-infected samples. Heterochromatic regions of the genome, such as centromeres, showed a lack of transcription in all samples (Figure 2C), suggesting no chromosome-level biases during Herts/33 or LaSota infection.

### 3.2. Alternative Splicing Events of Virus-Infected Chicken

Although AS events occur in plants and animals, they differ in the relative proportion and frequency of AS types [57]. Intron retention (IR) events are the predominant AS events in plants, while in animals, exon skipping (ES) events are the most common. Stress has also been shown to induce changes in the number and type of AS events. For example, heat stress decreased the frequency of IR events, while increasing the alternate acceptor (AA) and alternate donor (AD) events in *Physcomitrella patens* [58].

**Figure 3:**
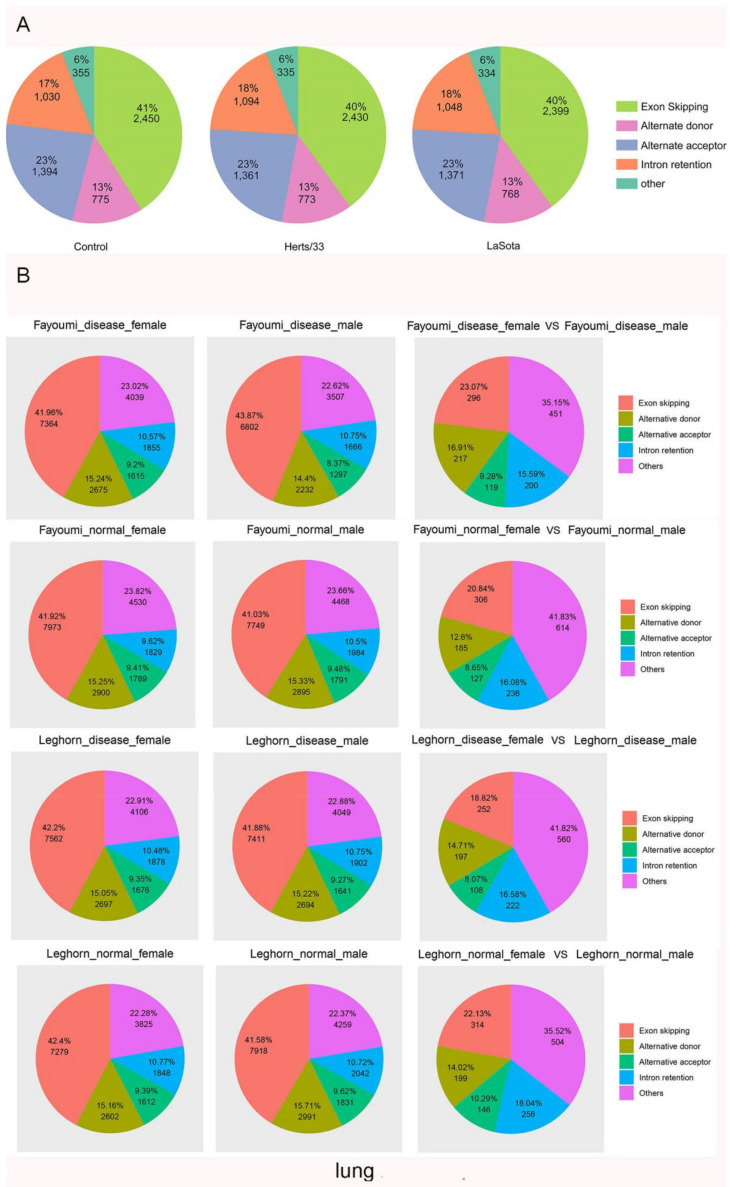

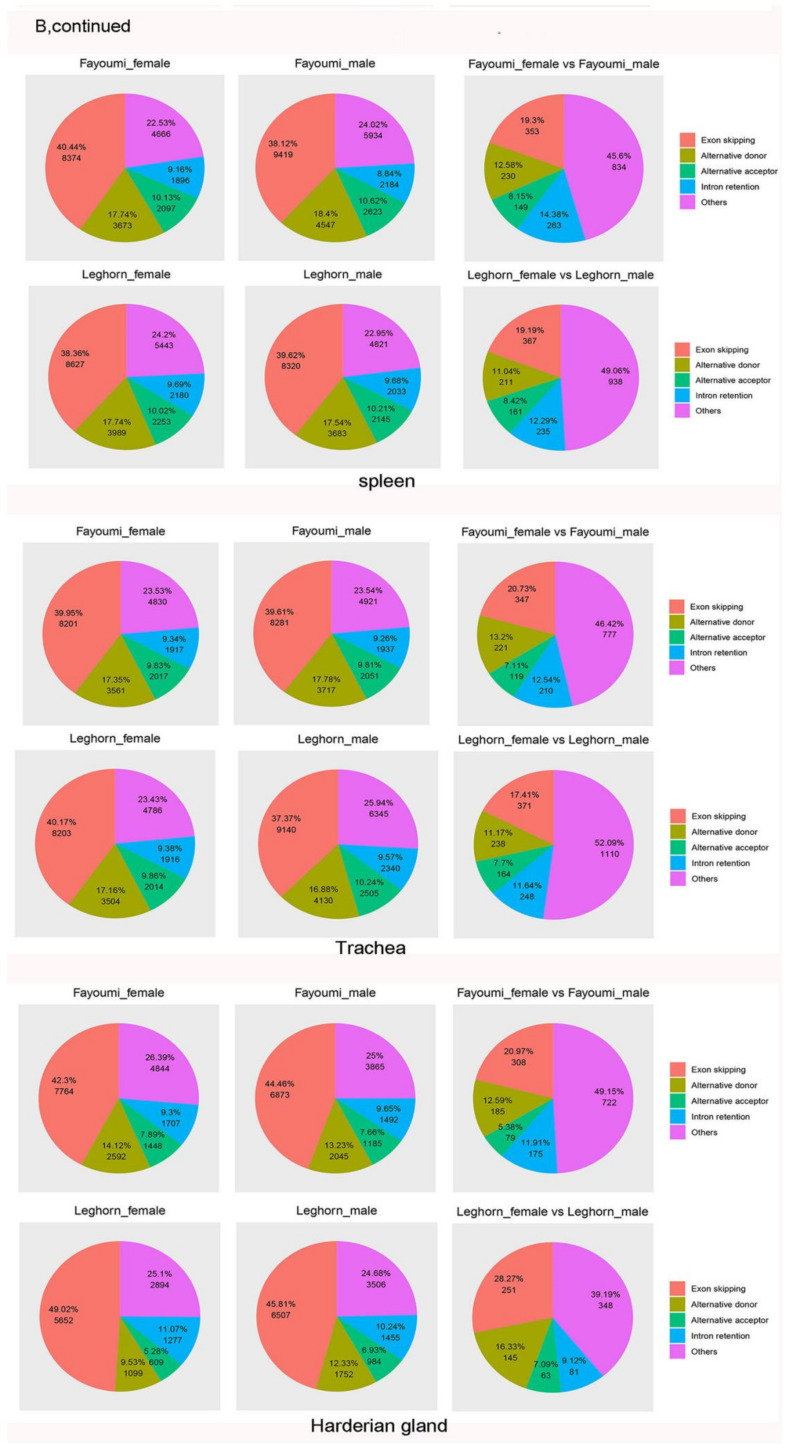

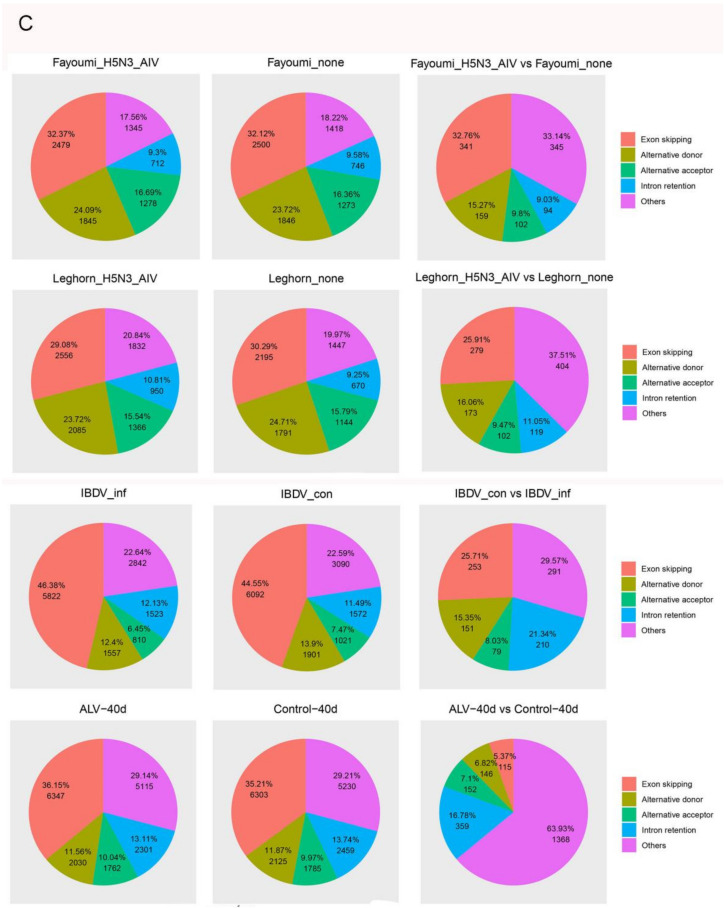
AS Landscapes in the control and virus-infected samples. (**A**) Frequency of AS types in the control, Herts/33-, and LaSota-infected CEF cells. (**B**) Frequency of AS types in lung, spleen, trachea and Harderian gland tissues and in different sex after NDV infection between Fayoumi and Leghorn chicken. In lung, “disease” indicates NDV infection group and “normal” indicates control group. (**C**) Frequency of AS types in control-, ALV- and IBDV-infected chickens and frequency of AS types in H5N3 infection between Fayoumi and Leghorn chicken. “IBDV_inf” indicates IBDV infection group and “IBDV_con”indicates control group. “ALV-40d” indicates ALV infection group and “Control-40d” indicates control group. Pairwise comparison pie charts generated using chi-square test between control group and infection group or between different sex. (χ^2^ test, *p* < 0.01). Gene annotations were obtained from Ensembl.

To analyze the AS landscape of Gallus gallus and determine what effects viral infection has, we used the Alternative Splicing Transcriptional Landscape Visualization (ASTALAVISTA) tool and categorized the AS events [54]. The AS events were categorized as: (1) Intron Retention (IR), (2) Exon Skipping (ES), (3) Alternative 3′ Acceptor Site (AA), (4) Alternative 5′ Donor Site (AD), and (5) other events. The “other events” are complex AS events comprising duplicated IR, AA, AD, and ES events or combinations of different AS events. Of the 21,490 intron-containing, multi-exonic genes detected in our study, 8911 genes (42%) were alternatively spliced. The splice junctions of these genes were used to identify, extract, and classify the AS events. In total, we identified 6004 AS events in the control group, 5993 AS events in Herts/33 infected group and 5920 AS events in LaSota (Figure 3A). Among the different AS types, ES events predominated (2450, 41%), followed by AA (1394, 23%), IR (1030, 17%), and AD (775, 13%). Approximately 355 events (6%) were classified as complex AS in the control group (Figure 3A). The overall distribution of AS events did not change after NDV infection in CEF cells (χ^2^ test, *p* > 0.05), implying there were no broad changes in the overall ratios of the different AS types in virus-stressed Gallus gallus in vitro.

We next expanded the analysis of AS events carried out in vitro to those in vivo by comparing the AS events in two inbred lines with different susceptibilities to NDV, including Leghorn (susceptible) and Fayoumi (resistant). Exon Skipping (ES) events were the most common AS type in all conditions, although the relative proportion of other AS events did vary by tissue type. Interestingly, the relative proportion of virus-responsive ES events was consistently lower in females than in males in all conditions we assessed (Figure 3B,C). At the whole transcriptome level, the lung of the nonchallenged Fayoumis at 10 dpi (days post infection) showed enrichment of transcripts associated with immune cells when compared to the number of immune cell-associated transcripts at 2 dpi, suggesting important immune-related development at this age which may play a role in this line’s viral resistance [44]. To better understand the possible underlying mechanism for this resistance, we analyzed the AS events in lung at 10 dpi in both Fayoumi and Leghorn.In Fayoumi, a comparison of AS events in normal versus diseased lungs revealed that ES events were consistently the most common, followed by AD, AC and IR in both diseased males and females. In Leghorn, ES events were also the most predominant, followed by AD, IR and AC (Figure 3B).

In spleen tissue, a comparison between NDV-infected and non-infected groups showed that NDV challenge induced a large amount of differential gene expression events, with more changes occurring at 2 dpi than at 6 dpi in both lines [45]. We therefore focused most of our analysis on AS events occurring at 2 dpi for this tissue. In both Fayoumi and Leghorn, ES events were predominant, followed by AD, AC and IR in males and females (Figure 3B). Although shared pathways were seen in the Fayoumi and Leghorn lines, each line showed unique responses as well. In particular, the downregulation of collagen and the activation of eukaryotic translation initiation factor 2 signaling in the Fayoumis relative to the Leghorns at 2 days post infection may contribute to the resistance phenotype seen in the Fayoumis.

Next, we analyzed the AS events in trachea tissue at 2 dpi [46]. In both Fayoumi and leghorn, ES events were predominant, followed by AD, AC and IR in males and females (Figure 3B).

The Harderian gland is a unique lymph tissue behind each eye of chickens. Fayoumi chickens had significantly more detectable viral transcripts in the Harderian gland at 2 dpi than the Leghorn but few genes were differentially expressed between the challenged and nonchallenged chicken at 2 dpi [47]. At 2 dpi, Fayoumi ES events were predominant, followed by AD, IR and AC in males and females. In Leghorn, ES events were predominant, followed by AD, AC and IR in males. In females, ES events were predominant, followed by IR, AD and AC (Figure 3B). Overall, these results indicated that sex may play an important role in AS events during NDV infection.

To determine whether another avian virus infection changed the AS events in Gallus gallus, we further identified and analyzed the AS events during infection with other avian viruses. In Fayoumi, ES events were predominant, followed by AD, others, IR and AC during H5N3 infection. The proportion of ES events decreased and the proportion of other events increased when comparing infected with uninfected samples (χ^2^ test, *p* = 6.758198 × 10^−33^). In Leghorn, ES events were predominant, followed by AD, other, AC and IR during H5N3 infection. The proportion of ES events decreased and the proportion of others increased when comparing infected with uninfected samples (χ^2^ test, *p* = 8.101033 × 10^−41^) (Figure 3C). In IBDV, ES events were predominant, followed by other, AD, IR and AC during IBDV infection. Once again, the proportion of ES events decreased and the proportion of other events increased when comparing infected with uninfected samples (χ^2^ test, *p* = 5.460467 × 10^−37^) (Figure 3C). In ALV infection, ES events were predominant, followed by other, AD, IR and AC. As with other viruses, the proportion of ES events decreased and the proportion of other events significantly increased when comparing infected with uninfected samples (χ^2^ test, *p* = 8.659221 × 10^−281^) (Figure 3C).

Taken together, these results show that the exon skipping mechanism is the predominant AS process in chickens during viral infection, and that infection can shift the relative proportions of other AS categories.

### 3.3. Virus-Modulated Alternatively Spliced Gene Cluster Analysis 

To identify alternatively spliced gene clusters that were differentially regulated by virus infection, we performed differential splicing analysis using the Cuffdiff program [50]. After statistical cutoffs (false discovery rate [FDR] < 0.05), we found a total of 844 genes that encode 2071 transcripts which were differentially spliced in NDV infected groups compared with the control (Table S11). Among the transcripts produced by differentially spliced genes, 1101 (53.1%) are known reference annotated transcripts, while the remaining 970 (46.9%) are novel transcripts or transcripts with annotations which are different from the Gallus gallus reference annotations (Table S8). There were 1571 transcripts detected from differentially spliced genes in the Herts/33 infection group and 500 transcripts in the LaSota infection group. Out of the 844 differentially spliced genes, 141 were found in comparisons of control against both Herts/33 and LaSota. In general, alternative splicing caused the production of a greater number of alternative transcripts in Herts/33 than in LaSota. For example, C2CD5 has five expressed isoforms during Herts/33 infection, but only two during LaSota infection. These results suggest virulent viruses may cause more extensive AS events than letogenic viruses.

**Figure 4:**
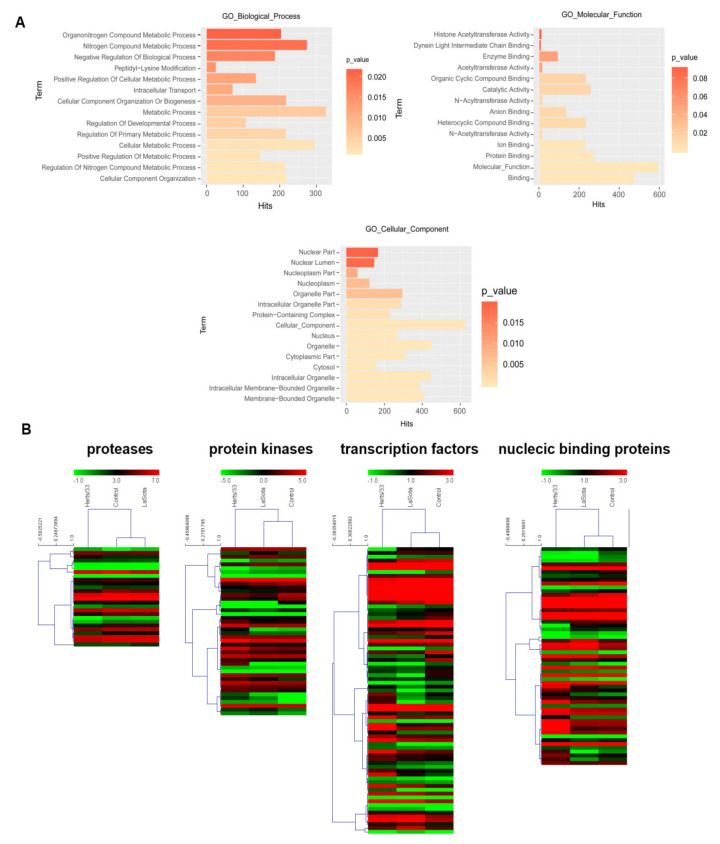
Virus-modulated differentially spliced gene clusters in NDV infected CEF cells. (**A**) GO biological processes, cellular components, and molecular functions enriched (The corrected P value with FDR < 0.05) among differentially spliced genes in Herts/33 and LaSota infection compared with control. The hits indicate that the number of genes corresponding to the enriched GO terms. (**B**) Hierarchical heatmaps of expression profiles of the AS transcripts encoding putative protease, protein kinases, transcription factors and nuclecic binding proteins in control, Herts/33 and LaSota infection. Node heights of the gene clusters are displayed above the gene trees. Colors represent log_2_FPKM expression values of the corresponding alternatively spliced transcripts.

We next examined the putative functions enriched among the 844 differentially spliced genes using GOEAST. Gene set enrichment analysis revealed significantly enriched (FDR < 0.05) terms related to metabolism (Figure 4A). Through the intersection of GO, PANTHER and KEGG functional annotations associated with immunity, we identified 218 alternative splicing genes, including 11 protein kinases, eight protein kinase receptors, 20 transcription factors, 12 proteases, 19 nucleic acid binding proteins, two splicing factors and seven RNA polymerases. We also found novel splice variants among key defense genes such as MAPK12, NFATC3, CXCL12, PLK3 and PIK3R1. Further, hierarchical cluster analysis of selected defense-related gene clusters showed discernable patterns of coregulation among the splice variants during Herts/33 infection and in the control group, compared with LaSota (Figure 4B). Taken together, these analyses identified AS events and splice variants in several defense-related genes and greatly expand our knowledge about how chickens employ AS to respond to viral infection.

### 3.4. Dynamic Analyses of Alternative Polyadenylation in 3′ UTRs 

**Figure 5:**
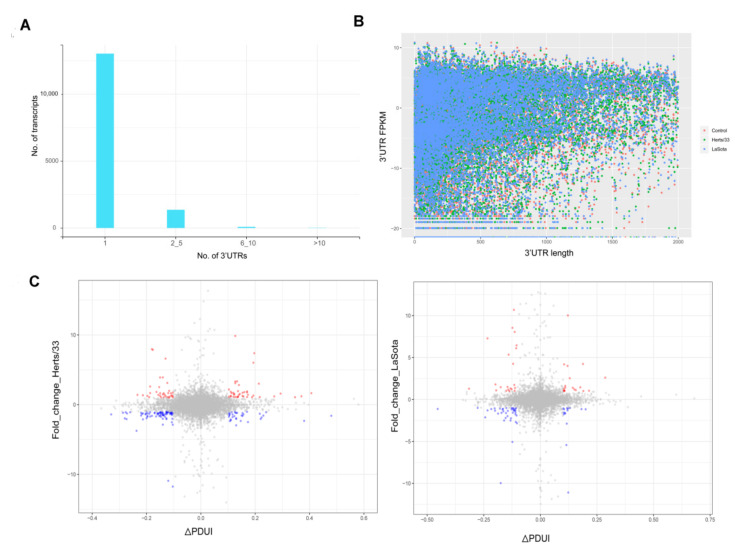
NDV infection induced alternate polyadenylation in CEF cells. (**A**)The number of transcripts in the Gallus gallus genome known to have 1, 2 to 5, 6 to 10 or more than 10 reported 3′ UTRs as obtained from Ensembl. (**B**) A plot comparing the length of 3′ UTR and the corresponding FPKMs across the control, Herts/33 and LaSota samples. (**C**) Percent distal poly-A usage index (PDUI) for transcripts and the corresponding FPKM values after NDV infection in CEF cells are shown. Transcripts falling below the PDUI threshold score of ±0.1 with expression levels below 1 are shown in grey. Increased expression is highlighted red and decreased expression is highlighted blue.

Different isoforms of a gene often vary in the length of their 3′ untranslated regions (3′ UTRs). The dynamic usage of 3′ UTRs is mediated through alternative polyadenylation (APA) and plays an important role in post-transcriptional regulation under diverse physiological and pathological conditions [59,60,61]. In order to determine whether APA plays any role in response to viral infection in chickens, we first analyzed the 3′ UTRs of annotated transcripts from the Ensembl database. As shown in Figure 5A, the majority of transcripts in Gallus gallus have only one 3′ UTR. Comparison of the different expression levels of transcripts with multiple possible 3′ UTRs in infected versus uninfected samples did not yield significant differences (Figure 5B).

The 3′ UTR length of transcripts can be regulated through proximal or distal poly-adenylation signals (poly-A signals). Typically, proximal poly-A sites result in smaller UTRs while distal poly-A sites cause longer UTRs. We calculated a percentage of the distal poly-A site usage index (PDUI) score to compare the relative UTR length versus expression using the DaPars algorithm (Tables S9 and S10) and found that transcripts with longer UTRs generally showed more differential regulation compared to transcripts with shorter UTR length (Figure 5C) [62]. This indicated that there was no distinct shift in the PDUI score in virus infected CEF cells, suggesting there was no obvious increase in the use of distal poly-A sites or the expression of transcripts with longer 3′ UTRs.

It has also been reported that AS and APA events may be linked, suggesting transcripts undergoing AS also have increased chances of undergoing APA [61]. In our data, we found that approximately 15% of transcripts that had APA also underwent AS in Herts/33 infection and approximately 16.7% of transcripts that had APA also underwent AS in LaSota infection. Gene ontology enrichment analysis of these transcripts revealed that genes with differential APA were enriched in categories associated with translation regulation, transcription regulation and catalytic activity (Tables S11 and S12). This result suggests that global APA events could be altered as a consequence of NDV infection.

### 3.5. Spliceosome during Virus Infection

**Figure 6:**
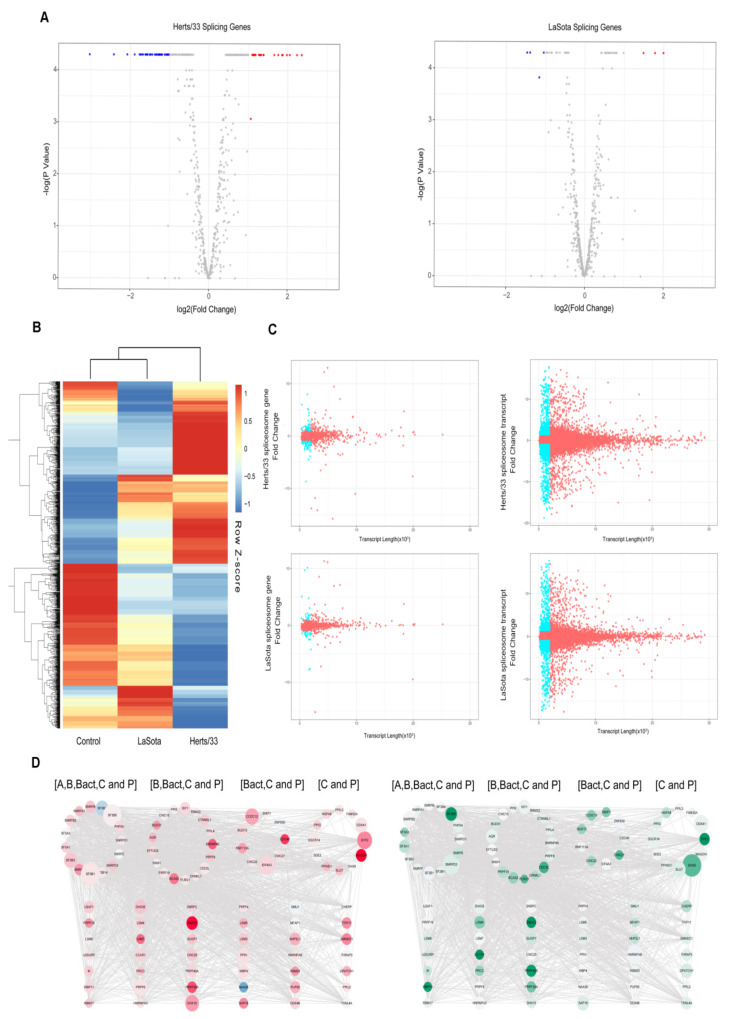
Spliceosome analysis upon NDV infection in CEF cells. (**A**) Volcano plot showing differential expression of genes associated with spliceosome function (|log_2_(fold_change)|> 1, *p* < 0.01). Red indicates up-regulation and blue indicates down-regulation. (**B**) A hierarchical heat map showing transformed expression values for the spliceosome transcript variants. Red indicates up-regulation and blue indicates down-regulation. (**C**) Plots showing length versus fold change in expression for each of the transcripts of the spliceosome genes under Herts/33 or LaSota infection. The dots in blue represent transcripts less than 2000 bp long and with the differential expression value of more than 1 or less than 1. (**D**) A, B, Bact, C and P (as marked) represent the five major of stages of spliceosome assembly and action. The size of each node was defined based on the length of the transcript that was maximally upregulated (in the top panel) or maximally downregulated for each of the genes (lower panel). Red color in the top panel identifies fold change greater than 1 in expression and green color in the lower panel identifies fold change less than 1 in expression. The expression data used to color and size the nodes were from Herts/33- infected cells.

We next examined the spliceosome complex in chicken after NDV infection to look for viral-induced perturbations in the splicing machinery. Approximately 10% (70/703) of the genes associated with spliceosomes had significant changes after NDV infection. Among these genes, 63 were significantly differentially expressed after Herts/33 infection, including 25 upregulated and 38 downregulated (Log2Foldchange > 1, *p* < 0.01). Interestingly, HSPA5 and MCM3 were most significantly changed (Figure 6A). Herts/33 infection, on the other hand, only caused the upregulation of three genes and downregulation of four genes (Log2Foldchange > 1, *p* < 0.01) (Figure 6B). We next compared the expression of the spliceosome-associated genes at the transcript level, revealing substantially higher differential splicing of these genes in both Herts/33 and LaSota infected CEF cells (Figure 6B). The list of transcripts associated with spliceosome genes and their corresponding expression levels is shown in Table S13. The range of transcript expression varied between ~9.5 fold downregulated to ~13 fold upregulated (Figure 6C).

We also analyzed the length of the genes involved in the spliceosome after NDV infection and compared the relationship between the length of these genes and their expression. There are 121 differentially expressed long transcripts (length ≥ 2000, q value < 0.05) and 72 differentially expressed short transcripts (length < 2000, q value < 0.05) comparing Herts/33 infection to control. In LaSota infection, there were 38 differentially expressed long transcripts (length ≥ 2000, q value < 0.05) and 20 differentially expressed short transcripts (length < 2000, q value < 0.05) identified (Figure 6D). These results indicated that the NDV infection did not cause significant changes in the transcript length of the genes associated with the spliceosome (Table S13).

### 3.6. Virus-Modulated Alternative Splicing of Splicing Factors

**Figure 7:**
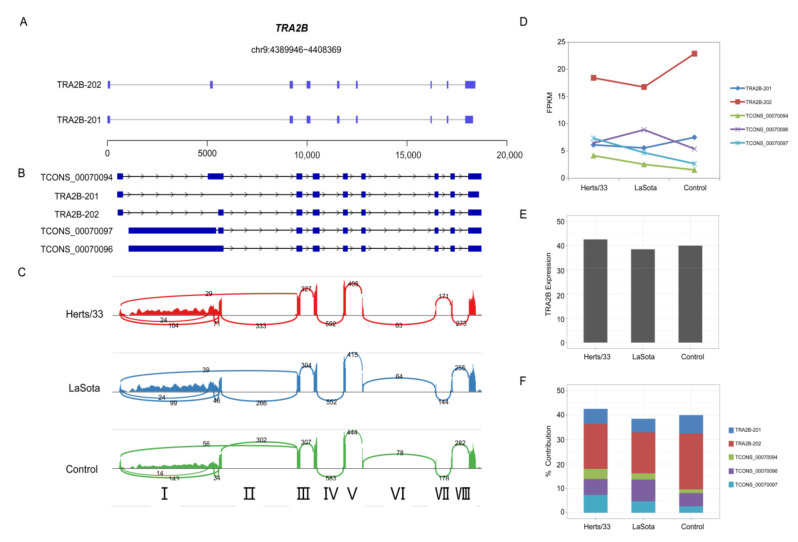
Virus-modulated alternative splicing patterns of CEF cells. (**A**) Reference gene annotations of TRA2B and its splice variants. The exon of TRA2B is indicated by the blue block. (**B**) Cufflinks-assembled transcript models of TRA2B splice variants. (**C**) Sashimi plots showing RNA-seq reads mapping to TRA2B locus in the control, Herts/33, and LaSota samples. Heights of the bars represent overall read coverage. Splice junctions supported by at least 10 split reads are displayed as loops. The number of reads corresponding to specific exon-exon junctions (shown as loops) is shown for each junction. Intron numbers Ⅰ through Ⅷ are indicated at the bottom of the panel. (**D**) Expression dynamics of TRA2B splice variants analyzed by RNA-seq in the control, Herts/33- and LaSota-infected CEF cells. (**E**) The FPKM values of TRA2B in control, Herts/33 and LaSota samples. (**F**) Percent contribution of TRA2B transcripts across the three groups, as observed in RNA-seq data.

In chickens, the mechanism of virus-induced AS likely involves spliceosomal proteins and splicing factors that are themselves alternatively spliced [63]. TRA2B is an important sequence-specific serine/arginine splicing factor which is involved in embryo and brain development in mice, and has been shown to cause diseases when dysregulated in humans. In humans, the different transcripts of TRA2B are produced by alternative skipping of its second exon. The Gallus gallus reference genome (V5) has two transcript annotations for TRA2B. The primary transcript, TRA2B-201, encodes a 189 amino acid protein while the second variant, TRA2B-202, encodes a 289 amino acid protein (Figure 7A). Expression of TRA2B-201 was below detectable levels in our analysis, while TRA2B-202 was the predominant splice variant with an FPKM of 22.8 in control. (Figure 7B). Expression of TRA2B-202 was significantly downregulated to 18.4367 FPKM after Hers/33 infection, and to 16.7597 FPKM after LaSota infection. (Figure 7C). However, we identified multiple new TRA2B splice variants in the Cufflinks transcript assembly that displayed varying expression patterns during virus infection (Figure 7B,C). A Sashimi plot visualization of the TRA2B splice variants revealed a substantial number of RNA-seq reads mapping to TRA2B intron I (Figure 7D).

## 4. Discussion

Chicken is a major agricultural product as well as an important model species. Although several studies have shown alternative splicing to be conserved between chicken and mammals, little was previously known about the effect of viral infection on AS in chickens [19,64]. Here, we have shown that viral infection stimulates a wide array of AS changes both in vitro and in vivo.

Viruses have been shown to modulate host gene expression in order to favor viral replication and evade antiviral responses. To this end, they have evolved mechanisms to affect cellular gene transcription, mRNA processing and nuclear export, mRNA decay and translation [65,66,67,68]. Several studies have indicated that viral factors can also target cellular spliceosome factors to alter the splicing of pre-mRNA, phosphorylation of SR proteins and stability, splicing and poly-adenylation of mRNAs. For example, the 3D protein of virus EV-71 can bind to a component of the U5 snNRP, PRPF8, and block pre-mRNA splicing and mRNA synthesis [69]. The Rev ULM motif of HIV can bind to the UHMs of U2AF65 and SPF45 to modulate the expression of HIV-1 genes, and Influenza V can bind to the U6 snRNA to inhibit pre-mRNA splicing [70]. In DENV infection, NS5 interacts with core components of the U5 snRNP particle, CD2BP2 and DDX23, in order to alter the inclusion to exclusion ratio of alternative splicing events, resulting in changes to mRNA isoform abundance of known antiviral factors [71].

Despite extensive knowledge about the effect of viral proteins on splicing in human and mouse systems, little is known about such systems in chickens. In this study, we demonstrated that AS events in chickens were significantly altered by NDV viral infection. The relative fractions of AS events were different between in vivo and in vitro experiments, possibly due to gene expression differences which are developmental stage- or tissue-specific. We also analyzed AS events during infection with ALV, IBDV and AIV. These results indicated that AS was a common phenomenon during viral infection in chickens, with ES consistently being the dominant AS category. Analysis of AS events in CEF cells showed similar patterns compared to earlier studies using DT 40 cells [63]. Although increased knowledge about the role of AS in viral responses in chickens has implications for the development of vaccines and other treatments, the exact mechanism of viral induced AS is still not fully understood.

Innate immunity is the first line of defense against pathogen invasion. In recent years, there has been growing evidence for the role of AS in shaping immune responses [72]. Different isoforms have been found to be key players in antiviral innate immunity, including pattern recognition receptors (TLRs, RIG-I, and MDA5), downstream signaling proteins (MyD88, MAVS, STING, TBK1, and IRF3), and effectors (IFN type I, IFNAR, cytokines, and chemokines) [73,74,75,76]. Additionally, a virus-induced, alternatively spliced isoform of TBK1 was shown to disrupt the interaction between RIG-I and MAVS and inhibit IFN-beta signaling [77]. Short isoforms of MAVS have been found to negatively regulate TLR3-mediated nucleic acid sensing and limit self-aggregation of the full-length MAVS protein, thereby preventing accidental antiviral innate immune signaling [78,79]. We discovered key immune-related gene clusters encoding protein kinases, nucleic acid binding proteins and splicing factors that are deferentially spliced during virus infection, revealing a possible link between alternative splicing and immune response. Further investigation into the exact mechanisms of AS alteration will be required to understand this process more fully.

Exon skipping, which is the most frequently detected form of AS in the human transcriptome, accounts for the largest share of infection-altered AS events in most viral infections, with the exception of DENV5 [9,71]. Interestingly, intron retention (IR), which was until recently considered a rare type of AS event in mammalian cells, was found to represent a substantial proportion (>20%) of infection-altered AS events in herpes simplex virus-1 (HSV-1) and DENV5-infected cells [36,71]. These observations align with recent findings that IR is actually a common AS event in mammalian cells, although the fate of intron-retaining mRNAs is not fully understood. Taken together with a recent report that influenza virus NS1 protein primarily binds intronic sequences, these findings suggest that some viruses may have evolved specific mechanisms to alter host gene expression through increased IR [80]. In our study, we found exon skipping was most frequently detected in the chicken transcriptome and accounted for the largest share of infection-altered AS events. This result suggests that avian viruses may alter chicken cell expression by changing ES events, although the specific mechanisms of the viral alteration of AS events will need to be further explored.

RNA-seq transcriptomic analysis is the most efficient method to analyze genome-wide changes in AS events. However, the accurate quantification of isoform abundance requires deep sequencing, with about 50 million paired-end reads of at least 75 bp recommended for the human transcriptome [81]. However, a serious limitation of Illumina RNA-seq is that it relies on short reads, so the resolution of exon connectivity and full-length isoform structure cannot be achieved definitively. In our study, some of the SRA data from Genebank used to analyze AS events were single-end reads with a length less than 100 bp. Therefore, the accuracy of the quantification of AS events from these datasets may be lower than usual. In the future, third-generation sequencing (TGS) technologies, such as the Pacific Biosciences (PacBio) and Oxford Nanopore (ON) technologies, could be used as alternative platforms for AS analysis. Moreover, this work mainly focuses on the genome-wide changes in AS events after virus infection in chickens but the predicted specific AS events occurred in the virus infections need to be verified in further study.

Studies have demonstrated that AS regulates host cell death pathways and host Type I and Type III IFN responses [82,83,84,85]. In addition, non-sense mediated decay (NMD), another post-transcriptional mechanism for gene expression regulation, mainly occurs under pathogen induced stress [86,87]. Alternative splicing (AS) coupled to nonsense mediated decay (NMD) is a post-transcriptional mechanism for regulating gene expression [88,89,90]. In this work, altered alternative splicing events were observed in genes involved in immune response and metabolism as well as genes related to splicing or transport. However, the role of the specific gene alternative splicing in antivrial or innante immunity remains unknown. The mechanisms of the specific gene alternative splicing in antiviral or innate immunity need to be further studied.

## 5. Conclusions

In this study, we used RNA-seq to examine virus-regulated gene expression at the post-transcriptional level in Gallus gallus. We have provided the first genome-wide analysis demonstrating that alternative splicing is induced by viral infections in chickens. Evidence from this study suggests that the mRNA splicing step can be modulated by virus infection to control the AS of transcripts involved in specific biological processes. However, the predicted specific AS events occurring in viral infections have not been verified and the role of the specific gene alternative splicing in antiviral or innate immunity remains unclear. These results are a key first step in understanding the role that alternative splicing plays in viral responses in chickens, and future work aimed at a deeper understanding of the underlying mechanisms of this process has potential implications for disease treatment and vaccination.

## Figures and Tables

**Table 1:** Statistical Summary of RNA-seq Mapping and Alignment.

	Total	B1	B2	B3	H1	H2	H3	L1	L2	L3
Mapped Reads										
Unique left (%)	299,224,346 (91.2%)	42,058,610 (90.3%)	42,726,173 (90.1%)	33,598,545 (91.9%)	24,791,424 (90.8%)	28,441,340 (91.8%)	29,097,409 (91.0%)	34,714,376 (91.4%)	31,484,844 (92.6%)	32,311,625 (91.3%)
Nonunique left (%)	28,796,379 (8.8%)	4,309,183 (9.7%)	4,705,478 (9.9%)	2,954,136 (8.1%)	2,516,090 (9.2%)	2,546,773 (8.2%)	2,863,948 (9.0%)	3,285,909 (8.6%)	2,525,643 (7.4%)	3,089,219 (8.7%)
Unique right (%)	285,044,438 (91.3%)	39,971,739 (90.3%)	41,096,311 (90.1%)	30,961,117 (91.9%)	23,667,626 (90.8%)	27,053,760 (91.8%)	27,702,817 (91.1%)	33,141,424 (91.4%)	30,353,878 (92.6%)	31,095,766 (91.3%)
Nonunique left (%)	27,808,310 (8.7%)	4,542,921 (9.7%)	4,495,046 (9.9%)	2,720,323 (8.1%)	2,397,429 (9.2%)	2,407,222 (8.2%)	2,716,835 (8.9%)	3,130,883 (8.6%)	2,429,221 (7.4%)	2,968,430 (8.7%)
Overall alignment	79.00%	86.80%	87.20%	85.80%	66.50%	66.70%	69.70%	82.70%	82.80%	82.60%
Total aligned pairs	293,168,743	41,610,655	42,736,163	31,659,213	24,439,859	27,508,308	28,424,075	33,979,545	30,741,417	32,069,508

B: blank; H: Herts/33; L: LaSota.

## Data Availability

RNA-seq raw sequence data of the CEF cells infected with Newcastle Disease Virus were obtained from our previous study. Other RNA-seq data, such as avian influenza virus (H5N3), infectious bursal disease virus (IBDV) and avian leukemia virus (ALV) were retrieved from the NCBI Short Read Archive database under the accession numbers SRP041937, ERP006921 and ERP017744. RNA-seq data from tissues infected with NDV, such as lung, spleen, trachea and Harderian gland were also retrieved from the NCBI Short Read Archive database under the accession numbers ERP024044, ERP023964, ERP021317 and ERP104372.

## Supplementary Materials

The following are available online at https://www.mdpi.com/article/10.3390/v13122409/s1, including Table S1. transcript_expression_differentialExpression.xlsx, Table S2. Ensemble_Ref_geneInfo, Table S3. Ensemble_Ref_transcriptInfo, Table S4. filteredGene.xlsx, Table S5. filteredIsoform.xlsx, Table S6. novel_gene.xlsx, Table S7. AS_Statistic.xlsx, Table S8. AlternativeSplicingGene.xlsx, Table S9. Herts33_DaPars_Res.xlsx, Table S10. LaSota_DaPars_Res.xlsx, Table S11. pantherChart-Herts_33.txt, Table S12. pantherChart-LaSota.txt, Table S13. Splicesome_GeneList.xlsx.
